# IFNα subtype-specific susceptibility of HBV in the course of chronic infection

**DOI:** 10.3389/fimmu.2022.1017753

**Published:** 2022-10-14

**Authors:** Xiaohong Xie, Zehra Karakoese, Dilhumare Ablikim, Julia Ickler, Jonas Schuhenn, Xiaoqing Zeng, Xuemei Feng, Xuecheng Yang, Ulf Dittmer, Dongliang Yang, Kathrin Sutter, Jia Liu

**Affiliations:** ^1^ Department of Infectious Diseases, Union Hospital, Tongji Medical College, Huazhong University of Science and Technology, Wuhan, China; ^2^ Department of Gastroenterology, Zhangzhou Affiliated Hospital of Fujian Medical University, Zhangzhou, China; ^3^ Institute for Virology, University Hospital of Essen, University of Duisburg-Essen, Essen, Germany; ^4^ Joint International Laboratory of Infection and Immunity, Huazhong University of Science and Technology, Wuhan, China

**Keywords:** IFNα subtypes, hepatitis B virus, persistent infection, hydrodynamic injection, IFN induction

## Abstract

Chronic hepatitis B virus (HBV) infection continues to be a major health problem worldwide and remains hard to be cured. Therapy with interferon (IFN) α is an important method for the clinical treatment of chronic hepatitis B. IFNα exhibits direct antiviral effects as well as immunomodulatory activities, which can induce sustained antiviral responses in part of the treated chronic hepatitis B patients. Numerous IFNα subtypes with high sequence identity between 76-96% exist which are characterized by diverse, non-redundant biological activities. Our previous studies have demonstrated that the clinically approved IFNα2 is not the most effective subtype for the anti-HBV treatment among all IFNα subtypes. So far very little is known about the IFNα subtype expression pattern during early HBV infection and the IFNα subtype-specific susceptibility during persistent HBV infection as well as its related cellular mechanism. Here we determined the *Ifna subtype* mRNA expression during acute and chronic HBV infection by using the well-established hydrodynamic injection (HDI) mouse model and we revealed a transient but strong expression of a panel of *Ifna subtypes* in the spleen of HBV persistent replication mice compared to HDI controls. Immunotherapy with distinct IFNα subtypes controlled chronic HBV infection. IFNα subtype-mediated antiviral response and immune activation were comprehensively analyzed in an AAV-HBV persistent infection murine model and murine IFNα2 was identified as the most effective subtype in suppression of HBV replication. Further analysis of the immune response revealed a strong immunomodulatory activity of murine IFNα2 on splenic and intrahepatic NK and T cell activation during persistent HBV infection. Taken together, our data provide IFNα subtype-specific differences in the antiviral and immunomodulatory effector responses and a strong expression of all IFNα subtypes in the spleen during persistent HBV infection in mice. This knowledge will support the development of novel immunotherapeutic strategies for chronic hepatitis B infection.

## Introduction

Hepatitis B virus (HBV) is a member of the *Hepadnaviridae* family. It is a hepatotropic, non-cytopathic, enveloped DNA virus that may cause acute and chronic liver inflammatory diseases. Although highly effective prophylactic vaccines are available, chronic HBV infections remain a major public health issue affecting approximately 296 million individuals with 1.5 million new infections every year (1). Persisting HBV predisposes to end-stage liver diseases, such as liver cirrhosis and hepatocellular carcinoma (HCC) and HBV is responsible for more than 800,000 deaths per year (1). Two types of antiviral therapies are currently approved for chronic HBV infection: nucleot(s)ide analogues (NUC), such as Entecavir and Tenofovir, and pegylated interferon alpha 2a/b (PEG-IFNα2a/b). NUCs target the viral reverse transcriptase leading to reduced viral replication. However, NUC treatments are not curative as they do not efficiently eliminate the HBV covalently closed circular DNA (cccDNA), which results in rebounding viremia after cessation of antiviral therapy (2). In contrast, IFNα2 therapy inhibits viral replication intermediates, blocks reinfection and improves clearance of infected hepatocytes through stimulation of immune cell responses (3, 4) and it is also able to efficiently reduce the cccDNA pool in HBV-infected hepatocytes (5, 6). It has been shown in patients who achieved long-term effective virological remission by NUCs that “adding-on” or “switching to” PEG-IFNα2a/b significantly increased the HBsAg loss rates to more than 20% (7, 8).

The early recognition of HBV by different pattern recognition receptors in hepatocytes is not completely understood so far. It was already shown that HBV is sensed by Toll-like receptor (TLR) 2, -3 and retinoic acid inducible gene I (RIG-I)/melanoma differentiation-associated protein 5 (MDA-5) signaling pathways (9); however, the expression of type I IFNs or IFN-stimulated genes (ISGs) is undetectable or even low (9–11). Thus, HBV was qualified as a “stealth virus” in comparison to other viruses like Hepatitis C virus (HCV) or human immunodeficiency virus (HIV). In contrast, early during HBV infection natural killer (NK) cells and natural killer T cells are activated (12–14), leading to the suggestion that HBV is able to evade the initial innate immune response. HBV can also suppress type I IFN-mediated antiviral immunity by reducing the production of type I IFN (9), inhibiting IFN-mediated downstream signaling, decreasing the surface expression of IFN receptors, attenuating the function and expression of ISGs, or impairing host innate and adaptive immune responses [reviewed in (15)]. So far, all reports on IFN induction during HBV infection *in vivo* or *in vitro* (9–11) showed low or no IFNα expression in HBV infection; however, detailed expression pattern of individual IFNα subtypes during different stages of HBV infection are still lacking.

Type I IFNs are among the first line of antiviral defense. In humans, the type I IFN family comprises IFNβ, IFNϵ, IFNκ, IFNω, and twelve IFNα subtypes (15). The human IFNα subtypes share similarities in structure, like the lack of introns or the length of the protein (161-167 amino acids), and their protein sequences are highly conserved (76 – 96% amino acid sequence identity) (16, 17). Despite binding to the same receptor consisting of the two subunits IFNAR1 and IFNAR2, the antiviral and antiproliferative potencies of the IFNα subtypes differ considerably (18–21). It is largely elusive, why different IFNα subtypes exhibit distinct effector functions. Different receptor affinities and/or interaction interfaces within the receptor have been discussed which may account for the observed variability in the biological activity (22, 23). Furthermore, the dosage, cell type, timing and the present cytokine milieu might further affect the type I IFN effector response (24). Previous studies *in vitro* and *in vivo* already revealed that other IFNα subtypes than human IFNα2 exhibited the highest anti-HBV potency (18). Human IFNα14 was identified as the most effective subtype for the suppression of HBV cccDNA transcription and HBeAg/HBsAg production. Importantly, IFNα14 treatment alone elicited an IFNα and IFNγ signaling crosstalk similarly to the combined usage of IFNα2 and IFNγ. This resulted in the induction of multiple potent antiviral effectors, which synergistically restricted HBV replication. Guanylate-binding protein 5 (GBP5), one of the most differentially expressed genes between IFNα14- and IFNα2-treated liver cells, was identified as a new HBV restriction factor (18). However, the IFN-mediated modulation of immune cell effector functions during chronic HBV infection still remains elusive.

In this study we aimed to investigate the mRNA expression of different *Ifna subtypes* in liver and spleen at different time points post HBV challenge in mice using the well-established hydrodynamic injection mouse model (HDI). During persistent HBV infection murine IFNα2 and IFNα11 strongly reduced HBV viremia, whereas murine IFNα4 and IFNα5 did not control chronic HBV infection. In addition, exogenous application of murine IFNα2 improved the host immune responses the most. Interestingly, two intervals of IFN therapy significantly increased the modulation of immune cell effector responses compared to short-term IFN treatment. Of note, similar effects of IFNα subtypes on CD8^+^ T cells were also observed in human PBMCs implying global IFN-mediated immunomodulatory effects. Here we clearly demonstrated IFNα subtype-specific differences in the antiviral and immunomodulatory effector responses during persistent HBV infection.

## Material and methods

### Mice

Male wildtype C57BL/6 mice were purchased from Vital River Laboratories Co., Ltd. (Beijing, China). All animals were bred and kept under specific pathogen-free (SPF) conditions in the Animal Care Center of Tongji Medical College (Wuhan, China).

### Virus and plasmid

BPS (genotype B persistent strain) plasmid was kindly provided by Prof. Xie from Fudan University. PSM2, a pUC19 vector-based plasmid harboring a head-to-tail-oriented HBV genome, was used to mimic acute-resolving HBV replication in mice after HDI. Recombinant adeno-associated virus 8 vector carrying 1.3 copies of HBV genome (rAAV8-HBV1.3) was purchased from Beijing FivePlus Gene Technology Co., Ltd. (Beijing, China).

### HBV replication mouse model

For hydrodynamic injection, 10 µg plasmids (pSM2 or BPS) in a volume of phosphate buffer saline (PBS) equivalent to 0.1 mL/g of the mouse body weight was injected through the tail vein within 5-8 seconds.

### Expression of murine IFNα subtypes and determination of IFN concentrations

Expression of murine (m)IFNα2, IFNα4, and IFNα5 were performed as previously described (25). To produce murine IFNα11, the cell line HEK293mIFNalpha11 was cultivated as described (26). All concentrated supernatants were tested for type I IFN concentration by a virus-free, cell-based bioassay using Mx/Rage 7 cells in comparison to commercially available recombinant mouse IFN (PBL assay science) (25, 26).

### HBV infection and mIFNα subtype treatment *in vivo*

A recombinant adeno-associated virus 8 vector carrying 1.3 copies of HBV genome (rAAV8-1.3HBV, 5.0 × 10^10^ viruses, 50 µL) was intravenously injected into the male C57BL/6 mice to induce HBV infection. After 4 weeks, the mice were intraperitoneally injected with 8000 U mIFNα subtypes (mIFNα2, IFNα4, IFNα5, or IFNα11) or left untreated (control) for 10 consecutive days. Blood samples were collected to dynamically monitor the characteristics of serum viremia.

### Serological assays

The levels of hepatitis B surface antigens (HBsAg) and HBeAg in the serum were determined by the corresponding ELISA kits (Kehua, Shanghai, China). A quantitative assay for HBsAg/HBsAb and HBeAg was conducted by commercial methods (Maglumi X8, SNIBE Co. Ltd., Shenzhen, China). HBV DNA copies were measured by a diagnostic kit for HBV DNA (Sansure, Changsha, China) using quantitative real-time PCR (qRT-PCR).

### Cell surface and intracellular staining of murine splenocytes and hepatocytes by flow cytometry

Cell surface and intracellular staining for flow cytometry analysis was performed as previously described (27, 28). The antibodies used in this study are listed in **Supplementary Table 1**. Cell debris and dead cells were excluded from the analysis based on scatter signals and Fixable Viability Dye eFluor 506 (eBioscience, San Jose, CA, USA). Fluorescence minus one (FMO) controls were used for all conditions. Data were acquired on a FACS Canto II flow cytometer and analyzed using FlowJo software (both BD Bioscience, Franklin Lakes, NJ, USA). Gating scheme and representative dot plots are shown in **Supplementary Figure 2**.

### RNA isolation

Total RNA was isolated from splenocytes and hepatocytes RNAiso Plus (Takara, Shiga, Japan). Isolated RNA was dissolved in RNase-free water and stored at -80°C.

### Real-time-PCR

Real-time-PCR (RT-PCR) analysis for the quantification of murine *Ifna subtypes* mRNA was performed using One Step SYBR^®^ PrimeScript™ RT-PCR Kit II (Takara) on the iCycler real-time amplification system (Bio-Rad, Hercules, CA, USA). The quantitative mRNA levels were determined by using CFX Manager™ Software v3 (Bio-Rad, Hercules, CA, USA) and were normalized to *β-actin* mRNA expression levels. Sequences of oligonucleotides are shown in **Supplementary Table 2**.

### Isolation of PBMCs

PBMCs were isolated from each blood sample by density gradient centrifugation. For this purpose, 9 ml of EDTA-whole blood mixed with RPMI 1640 supplemented with 100 U/ml penicillin and 100 µg/ml streptomycin was layered on Pancoll solution (Pan Biotech, Aidenbach, Germany) and centrifuged at 900 x g for 35 minutes with brakes off. Then, the PBMCs (interphase) were transferred to a new 50 ml tube and washed twice with RPMI 1640 medium supplemented with penicillin/streptomycin. Cryostocks with 1x10^7^ PBMCs/ml were prepared in fetal calf serum (FCS) (Sigma Aldrich, St. Louis, MO, USA) supplemented with 10% DMSO.

PBMCs were thawed one day prior to experiments. Up to 90% of viable cells were cultivated in RPMI 1640 with 10% FCS, 100 U/ml penicillin, 100 µg/ml streptomycin, 2 mM L-glutamine, and 10 mM HEPES. Cells were incubated at a density of 1x10^6^ cells/ml over night at 37°C, 5% CO_2_.

### Stimulation with different human IFNα subtypes

Human IFNα subtypes were produced and purified as previously described (29). Briefly, recombinant IFNs were expressed in *E. coli* after M13 phage transduction. To harvest the proteins, the bacteria were pelleted, the protein-containing inclusion bodies were denatured by sonication, dissolved in 6M guanidin-hydrochlorid, and refolded in arginine. The recombinant proteins were further purified by ion exchange chromatography and size exclusion chromatography, specificity and purity of the proteins were verified after each step *via* an SDS gel. By phase separation of the products with Triton X-114, remaining endotoxin was removed from the solution. Endotoxin levels were tested using ToxinSensor (GenScript, Piscataway, NJ, USA) and are below 0.25 EU/mL. The activity of each subtype was determined using the human ISRE-Luc reporter cell line, a retinal pigment epithelial cell line transfected with a plasmid containing the Firefly Luciferase gene, stably integrated under control of the IFN-stimulation-response element (ISRE). Following stimulation with type I IFNs, chemiluminescence can be detected and used to calculate the respective activity in units against commercially available IFNα (PBL assays sciences, Piscataway, NJ, USA) (20).

### Stimulation with SEB

PBMCs from healthy individuals were stimulated with 200 ng/ml Staphylococcal enterotoxin B (SEB) (Merck, Darmstadt, Germany) in the presence of 20 U/ml IL-2 (Miltenyi Biotec, Bergisch Gladbach, Germany) and treated with 2000 U/ml IFNα subtypes, or without IFN (-IFN) for 4 days. Then, PBMCs were re-stimulated with 5 µg/ml SEB and incubated in presence of antibodies against the co-stimulatory molecules CD28 (9F10, BioLegend, San Diego, CA, USA) and CD49d (CD2.2, BioLegend) at 37°C for 6 h. Brefeldin A with a final concentration of 5 µg/ml was added after 1 h of stimulation. Cells were immediately used for flow cytometric analysis.

### Cell surface and intracellular staining of PBMCs by flow cytometry

For surface staining, cells were washed once with FACS buffer (PBS containing 0.1% BSA and 0.02% sodium azide) and cells were incubated for 15 min with the antibody mixture in FACS buffer. Cell surface staining was performed using the antibodies listed in **Supplementary Table 1**. The Fixable Viability Dye eFluor™ 780 (eBioscience) was used to exclude dead cells from the analysis. Cells were washed with FACS buffer and fixed with Fixation Buffer (BioLegend). Cells were washed twice with Intracellular Staining Perm Wash Buffer (BioLegend) and incubated for 20 min with intracellular targeting antibodies listed in **Supplementary Table 1** in Intracellular Staining Perm Wash Buffer. Cells were washed again twice with Intracellular Staining Perm Wash buffer, collected in FACS staining buffer, and stored at 4°C until acquisition. Samples were acquired with BD FACSymphony™ A5 Cell Analyzer and data were analyzed using FACSDiva and FlowJo Version 10.8.

### Statistical analysis

Experimental data were reported as means +SEM. All statistically significant differences between the all groups were analyzed using Friedman test and Dunn’s multiple comparison test (human samples) or one-way ANOVA (mouse samples). Statistical analyses were performed using GraphPad Prism software v8 (GraphPad, San Diego, CA, USA).

## Results

### All *Ifna subtypes* are strongly induced during persistent and self-resolving HBV infection *in vivo*

**Figure 1:**
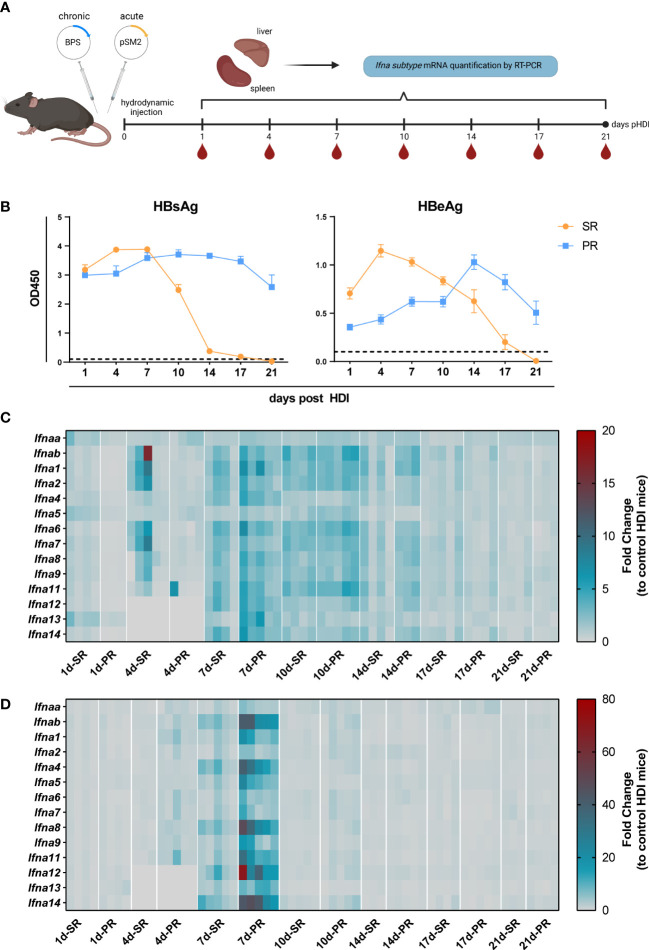
The mRNA expression profile of Ifna subtypes of HBV replication mice models. **(A)** Male C57BL/6 mice were hydrodynamically injected with pSM2 and BPS plasmids to establish HBV self-resolving (SR) and persistent replication (PR) mice models respectively. Mice hydrodynamically injected with PBS were used as HDI controls. Peripheral blood, liver, and spleen tissue samples were collected to extract total RNA at 1, 4, 7, 10, 14, and 21 days after injection. The mRNA expression of mIFNα subtypes was detected by RT-PCR. Five mice were sacrificed in each group at each indicated timepoint. Created with BioRender.com. **(B)** Levels of HBsAg (left) and HBeAg (right) in serum at indicated time points. **(C)** The mRNA expression profile of IFNα subtypes in liver and **(D)** spleen tissues represented as fold changes compared to HDI control. **(C, D)** Three to five mice were analyzed at the respective timepoint and individual mice are depicted as boxes.

So far, it remains unknown whether HBV infection induces the expression of certain types of *Ifna subtypes*, and how the kinetic of *Ifna subtype* mRNA expression differs between self-resolving (SR) and persistent-replicating (PR) HBV infection. Thus, we utilized the well-established HBV hydrodynamic injection (HDI) mouse model using the two different plasmids (pSM2 and BPS) to mimic acute and chronic HBV infection in C57BL/6 mice (**Figure 1A**). As previously shown (30, 31), application of pSM2 plasmid resulted in HBsAg and HBeAg clearance at 2-3 weeks post HDI, whereas hydrodynamic injection of BPS led to persistent expression of HBsAg and HBeAg (**Figure 1B**). Next, we analyzed the mRNA expression of all murine *Ifna subtypes* in liver (**Figure 1C**) and spleen (**Figure 1D**) in comparison to control HDI (PBS) mice at different time points post HDI. Of note, at 4 days post HDI significant upregulation of some murine *Ifna subtypes* (*Ifnab, Ifna1, Ifna2, Ifna6, Ifna7, IFNa8*, and *Ifna9)* was already detected in the liver of SR mice, whereas at 7-10 days post HDI up to 7-fold increase in the expression of all *Ifna subtypes* was observed in both SR and PR mice compared to control mice, which started to decline at day 14. Interestingly, in the spleen (**Figure 1D**) an upregulation of *Ifna subtype* mRNA was only observed at 7 days post HDI, and was only markedly increased in PR but not SR mice. The expression of distinct IFNα subtypes (*Ifnab, Ifna4, IFNa8, Ifna12*, and *Ifna14)* was utmost induced in the spleen. Thus, using the HDI mouse model, we demonstrate that a transient but strong type I IFN mRNA expression was induced in the spleen of mice injected with persistent-replicating HBV.

### Therapeutic treatment with distinct IFNα subtypes efficiently controlled persistent HBV infection in the HDI mouse model

**Figure 2:**
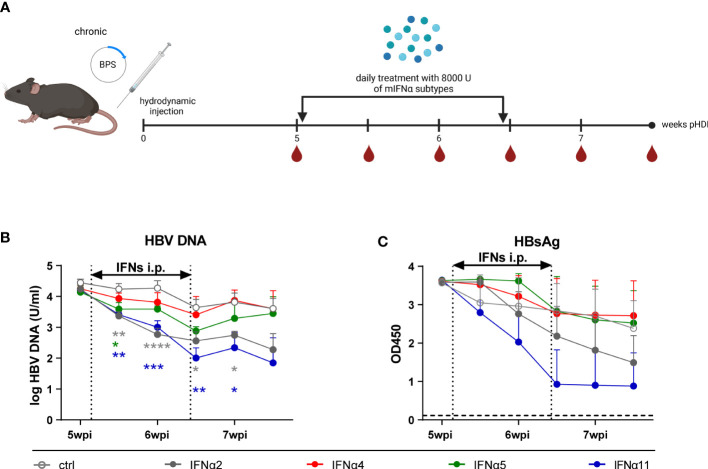
The virus levels of HBV persistent replication (PR) mice after IFNα subtypes administration. **(A)** The BPS plasmid hydrodynamically injected mice were intraperitoneally injected with 8000 U mIFNα subtypes for 10 consecutive days at 36 days post hydrodynamic injection. Created with BioRender.com. Additionally, blood samples were collected before, during, and after mIFN treatment to dynamically monitor the characteristics of serum viremia shown as **(B)** log HBV DNA and **(C)** HBsAg (OD at 450nm). Mean values ± SEM are shown for ctrl, n=4; IFNα2, n=5; IFNα4, n=5; IFNα5, n=5; IFNα11, n=5. Statistical analyses between the treated groups and the untreated group were done by one-way ANOVA. *p<0.05; **p<0.01; ***p<0.001; ****p<0.0001.

In previous experiments using the HDI model of acute HBV infection in Balb/c mice, we already reported subtype-specific effector functions with murine IFNα4 and IFNα5 controlling HBV infection the most. In addition, strong immunomodulatory effects of murine IFNα4 and IFNα5 on T and NK cells were reported in spleen and liver (32). Thus, we aimed to elucidate the role of murine type I IFNs during chronic HBV infection. As depicted in **Figure 2A**, hydrodynamic injection of the BPS plasmid was performed to establish chronic infection (33) and IFN-treatment was started at day 36 post HDI on 10 consecutive days. In addition to IFNα4 and IFNα5, which were previously used during acute HBV infection, we also applied murine IFNα2 and IFNα11, which had a strong immunomodulatory effect on CD4^+^ T cell responses *in vitro* (data not shown) or antiviral activity in acute and chronic Friend retrovirus infection *in vivo*, respectively (34, 35). Human IFNα14, which was the most effective subtype in controlling HBV infection *in vitro* (18), was not included here due to unclear cross-species activity of human type I IFNs in mice. In contrast to acute HBV infection, therapeutic treatment with IFNα4 and IFNα5 did not inhibit HBV replication as measured by HBsAg and HBV DNA levels. However, therapeutic application of IFNα2 or IFNα11 significantly reduced HBV viral loads as shown by HBV DNA and HBsAg levels (**Figures 2B, C**). IFNα11 treatment resulted in up to 40-fold decrease in HBV DNA in the serum of mice compared to untreated control mice. All mice failed to develop detectable HBs antibody (HBsAb) levels in the serum during the whole observation period (data not shown).

### Repeated therapeutic treatment approach with IFNα2 resulted in better control of HBV infection and improved antiviral immune responses in an AAV-HBV persistent infection mouse model

**Figure 3:**
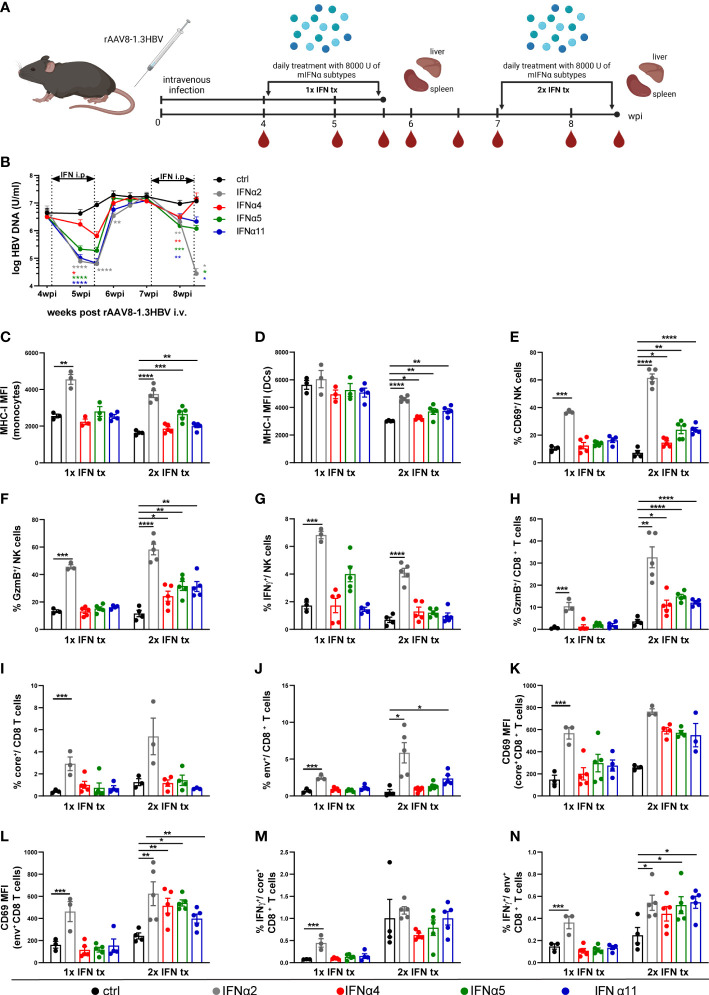
Distinct antiviral and immunomodulatory activities of different IFNα subtypes during HBV infection. **(A)** Male C57BL/6 mice were intravenously injected with rAAV8-1.3HBV to establish HBV infection. After 29 days post infection mice were treated by intraperitoneal injection with 8000 U mIFNα subtypes for 10 consecutive days. Blood samples were collected before, during, and after mIFN treatment to dynamically monitor the characteristics of serum viremia. One day after the treatment with mIFNα subtype, the mice were sacrificed to freshly separate the intrahepatic lymphocytes (IHLs)for immune function analysis by flow cytometry. For a second group of mice the same procedure was additionally repeated at 50 dpi. Created with BioRender.com. **(B)** The HBV DNA levels after mIFNα subtypes administration were determined by RT-PCR (ctrl, n=9-14; IFNα2, n=8-12; IFNα4, n=10-15; IFNα5, n=10-15; IFNα11, n=11-16). **(C, D)** The MHCI expression (MFI) of intrahepatic monocytes and dendritic cells was analyzed by flow cytometry **(E–G)** The CD69/GzmB/IFNγ expression of intrahepatic NK cells were analyzed by flow cytometry **(H)** The GzmB expression of intrahepatic CD8^+^ T cells was analyzed by flow cytometry **(I, J)** Frequencies of HBV core- and env-specific CD8^+^ T cells were detected by Tetramer staining **(K, L)** The expression of CD69 on core-specific and env-specific CD8^+^ T cells were analyzed by flow cytometry. **(M, N)** IHLs were stimulated with HBcAg epitope peptide (core93) or HBsAg epitope peptide (env208) for 5 h *in vitro*. Intracellular staining is performed, and the frequencies of IFNγ+ CD8 T cells are shown. **(C–N)** Individual mice are depicted as dots. Mean values ± SEM are shown for 1x ctrl, n=3; 1x IFNα2, n=3; 1x IFNα4, n=3-5; 1x IFNα5, n=3-5; 1x IFNα11, n=4; 2x ctrl, n=3-5; 2x IFNα2, n=3-5; 2x IFNα4, n=4-5; 2x IFNα5, n=4-5; 2x IFNα11, n=3-5. Statistical analyses between the treated groups and the untreated group were done by one-way ANOVA. *p<0.05; **p<0.01; ***p<0.001; ****p<0.0001.

Although, the HDI mouse model is a suitable model to determine immune responses against HBV, the model has also limitations like the lack of inflammation in mice with persistent HBV replication, general absence of cccDNA and low intrahepatic transfection efficiency, which is much lower compared to HBV-infected patients (reviewed in (36)). Therefore, we applied an hepatotropic AAV containing 1.3-fold HBV genome, which leads to HBV replication, secretion of infectious particles and cccDNA formation (36). To study the antiviral effects of murine IFNα2, IFNα4, IFNα5, and IFNα11 on persistent viral infection using a replicating virus *in vivo*, mice received rAAV8-1.3HBV i.v. and 4 weeks post infection IFN-treatment was performed on 10 consecutive days. One day later, half of the mice were sacrificed for analysis, whereas the other half received a second round of IFN-therapy starting at day 50 post infection to determine if repeated IFN therapy could further improve the outcome of the antiviral treatment (**Figure 3A**; 1xIFN tx; 2xIFN tx). Again, as seen in HDI mouse model (**Figure 2**), first treatment interval with IFNα2 and IFNα11 markedly reduced HBV DNA levels (up to 135-fold) in comparison to untreated controls (**Figure 3B**), but treatment interruption resulted in complete viral rebound. A second IFN-treatment interval further reduced HBV DNA level after IFNα2 application more than 420-fold compared to untreated controls, whereas second IFN-treatment with IFNα4, IFNα5, and IFNα11 marginally reduced viral titers potentially due to IFN desensitization. However, no significant effects on HBsAg and HBeAg levels after treatment with the different IFNs were observed (**Supplementary Figure 1**). All mice failed to develop detectable HBsAb levels in the serum during the whole observation period (data not shown). The serum alanine aminotransferase (ALT) and aspartate aminotransferase (AST) levels did not significantly change in IFN-treated mice AST (**Supplementary Figure 1**).

### Exogenous application of murine IFNα2 significantly improved immune responses in chronically HBV-infected mice

As the beneficial outcome of an IFNα2a/b therapy in patients depends on direct antiviral and immunomodulatory activities, we next investigated host immune responses in liver (**Figure 3**) and spleen (**Supplementary Figure 3**) after the first and second IFNα subtype treatment interval during persistent HBV. The total numbers as well as percentages of NK cells and T cells did not noticeably change during IFN-treatments in the liver (**Supplementary Figures 3A–C**). Especially after the second round of IFN treatment MHC class I expression per cell (mean fluorescence intensity, MFI) was significantly increased on monocytes and dendritic cells with IFNα2 increasing the surface expression the most (**Figures 3C, D**). Similar results were also observed for the percentages of activated (CD69^+^) and GzmB-expressing NK cells (**Figures 3E, F**). Interestingly, the frequencies of IFNγ-producing NK cells were solely increased after IFNα2-application, and the effect was stronger after the first IFN-interval compared to the second IFN-interval (**Figure 3G**). Next, we elucidated the impact of IFNα therapy on HBV-specific CD8^+^ T cell phenotypes and their effector functions. Percentages of CD8^+^ T cells expressing GzmB was remarkably enhanced after the second round with all different IFNs, but the strongest effect was again seen with IFNα2, which already improved GzmB expression after the first IFN-treatment interval (**Figure 3H**). Tetramer stainings to identify core-specific and env-specific CD8^+^ T cells revealed significantly higher percentages after one or two rounds of IFNα2-treatment, whereas the activation (CD69 MFI) on env-specific CD8^+^ T cells was significantly increased with all IFNα subtypes (2x IFN tx; **Figures 3I–L**). In *in vitro* peptide stimulation with core93 or env180 similar results on IFNγ-expressing env-specific CD8^+^ T cells were observed, indicating that 2 rounds of IFN treatment specifically improved env-specific CD8^+^ T cell responses (**Figures 3M, N**). Furthermore, we also analyzed the immunomodulatory effects of the IFNα subtypes on monocytes, DCs, NK, and T cells in the spleen at the same time points. Similar tendencies and higher percentages after IFNα2 treatment were observed, but the effects on the splenic immune responses were not significant (**Supplementary Figures 3D–P**).

To scrutinize if the observed immunomodulatory effects of the tested IFNα subtypes in the liver are further influenced by HBV itself, we treated HBV-uninfected mice with IFNα2, IFNα4, IFNα5, and IFNα11 for ten consecutive days and subsequently analyzed the immune response in the liver (**Supplementary Figure 4A**). Similar to the results observed after the first interval of IFN-treatment during persistent HBV infection, we detected higher frequencies of MHC-class I expressing monocytes and DCs (**Supplementary Figures 4B, C**), activated, GzmB and IFNγ expressing NK (**Supplementary Figures 4D–F**) and CD8^+^ T cells (**Supplementary Figures 4G–I**) only after therapeutic treatment with IFNα2. As we observed similar frequencies of activated immune cells and effector subsets in naïve and HBV-infected animals after 1xIFN tx, we suggest no direct HBV-mediated inhibition of IFN-responses (6) after 1xIFN tx. Taken together, these data further imply that, apart from its direct antiviral effect, IFNα2 stimulated antiviral effector functions of different immune cell subsets during persistent HBV infection likely contributed to the control of viral replication.

### IFN-stimulation of polyclonal triggered PBMCs from healthy individuals strongly modulated T and NK cell responses

**Figure 4:**
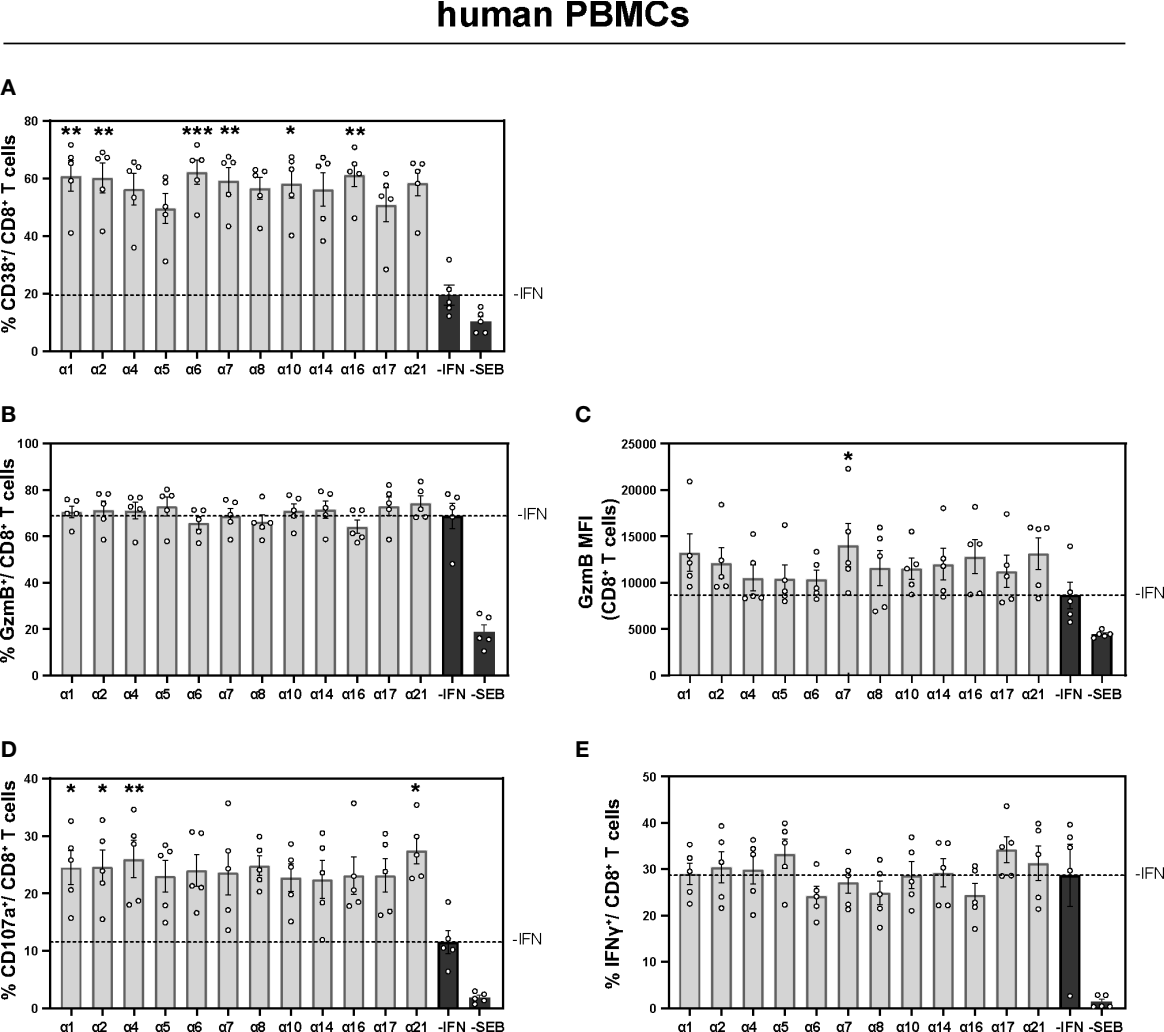
Expression of cytotoxic molecules by in vitro stimulated T and NK cells of PBMCs from healthy individuals PBMCs from healthy individuals were stimulated with 200 ng/ml SEB in presence of 20 U/ml IL-2 and treated with 2000 U/ml IFNα subtypes or without IFN (-IFN) for 4 days. PBMCs were re-stimulated with 5 µg/ml SEB and incubated in presence of antibodies against the co-stimulatory molecules CD28 and CD49d for 6 h. BFA was added after 1 h of stimulation. Flow cytometry was used to analyze T cell activation and cytokine expression. **(A)** Activation profile determined by the frequencies of CD38^+^ CD8^+^ T cells with or without stimulation in the presence or absence of the different IFNs. **(B, C)** Frequencies of GzmB-expressing CD8^+^ T cells and the GzmB expression per cell shown as MFI. **(D)** Frequencies of degranulating CD107a CD8^+^ T cells and **(E)** IFNγ expressing CD8^+^ T cells are shown. Mean values ± SEM are shown for **(A–C)** n=6 and **(D, E)** n=3. Statistical analyses between the treated groups within a cell population were done by using Friedman test and Dunn’s multiple comparison test. *, p < 0.05 **p<0.01; ***p<0.001.

To ensure that the observed immunomodulatory effects of murine IFNα2, IFNα4, IFNα5, and IFNα11 were not specific to mouse IFNs, we investigated the role of human IFNα subtypes on PBMCs of healthy individuals. Therefore, PBMCs were stimulated with staphylococcal enterotoxin B (SEB) in order to trigger polyclonal T cell activation (37) for 4 days in the presence and absence of the different human IFNs. At day 4 post stimulation the cells were re-stimulated with SEB and analyzed by flow cytometry. As shown in **Figure 4**, additional stimulation with IFNα subtypes increased the frequencies of activated CD38^+^ CD8^+^ T cells up to 3-fold ((-IFN: mean: 19.52%; IFNα6: mean 62.24%), and this effect was also significant after stimulation with human IFNα1, IFNα2, IFNα6, IFNα7, IFNα10, and IFNα21 (**Figure 4A**). Next, we analyzed effector functions of CD8^+^ T cells and we observed significantly higher percentages of CD107a-expressing CD8^+^ T cells and higher GzmB expression (MFI) after stimulation with IFNα1, IFNα2, IFNα4, IFNα21, and IFNα7, respectively (**Figures 4B–D**). However, no significant differences on human IFNγ^+^ CD8^+^ T cells were detected (**Figure 4E**).

Taken together, we could show that murine *Ifna subtype* mRNA was transiently induced in liver and spleen of the HDI mouse model. Furthermore, *in vivo* treatment with murine IFNα2 and IFNα11 strongly reduced HBV DNA and HBsAg level during chronic HBV infection using BPS HDI. Therapeutic treatment with IFNα2 strongly activated immune responses in the liver of chronic HBV-infected mice (rAAV8-1.3HBV), whereas repeated treatments with IFNs further improved host immune responses. These data clearly demonstrate IFNα subtype-specific differences in the antiviral and immunomodulatory response during chronic HBV infection.

## Discussion

In this study, we described that different murine IFNα subtypes show distinct antiviral activities in inhibiting HBV replication as well as inducing anti-HBV T cell and NK cell responses *in vivo* in a chronic HBV infection mouse model. The most effective subtype, murine IFNα2, demonstrated superior ability in inducing NK cell and CD8 T cell activation in both naïve and chronic HBV infected mice than the other tested IFNα subtypes. In line with the findings in the different used mouse models, we also observed that different human IFNα subtypes show distinct potencies in improving CD8^+^ T cell activation and effector functions *in vitro*. These results suggest that selecting an appropriate IFNα subtype to replace or combine the currently used human IFNα2 in HBV immunotherapy may achieve better antiviral effects for chronic HBV infection in humans.

HBV is traditionally described as a “stealth virus”, but it has been shown that HBV is sensed by different pattern recognition receptors (9); however, expression of innate immunity genes like *IFN*s or *ISGs* is rather low or even undetectable (9–11). Interestingly, the characteristic of poor induction of early type I interferon production is not associated with HBV persistence as shown in the HBV infected chimpanzee models (11). In contrast, we recently showed that simultaneous or prior activation of intrahepatic type I IFN signaling leads to HBV persistence in an HBV HDI mouse model (38). Here, we further characterized the expression kinetics of different IFNα subtypes in the liver and spleen post HBV exposure and compared their differences between acute-resolving and persistent HBV replication. In line with previous observations, we observed only weak upregulation of mRNA expression of certain IFNα subtypes in the liver of both acute-resolving HBV replication mice and persistent HBV replication mice. However, we observed that *Ifna subtype* mRNA expression was strongly upregulated in the spleen at early-stage post HBV exposure in HBV persistent replication mice, but not in acute-resolving HBV replication mice. This is, to our knowledge, the first characterization of early IFNα subtype expression in the scenario of chronic HBV infection. The results demonstrate that HBV may also trigger robust type I IFN responses; however, these responses might be associated with unfavorable outcome for HBV clearance.

Host innate and adaptive immune responses are very important to determine the outcome of HBV infection. NK cells represent the main effector population of the innate immune system in the liver that is able to recognize virus-infected hepatocytes. They secrete either IFNγ or TNFα to induce apoptosis in infected cells or directly eliminate these cells by the expression of granzymes, Fas ligand, or killer cell immunoglobulin-like receptors. In chronic HBV patients, altered phenotype and impaired function of NK cells were found (39). The IFNγ and TNFα production by NK cells are also strongly suppressed during chronic HBV infection (40). In adaptive immune responses, T cells play a fundamental role in HBV clearance and pathogenesis. Cytotoxic CD8^+^ T cells (CTL) can control viral infection by killing virus-infected cells through various effector molecules (Granzymes, TRAIL, Fas ligand). The non-cytopathic effector functions of CD4^+^ and CD8^+^ T cells like the production of antiviral cytokines (IFNγ, TNFα) are indispensable to control HBV infection (41, 42). During acute HBV infection, virus-specific CD8^+^ T cells were required for the control and elimination of HBV infection (43). Previous studies in HBV-infected chimpanzees also reported, that the depletion of CD8^+^ T cells during acute HBV infection resulted in remaining high viral titers (44), emphasizing their importance in viral control. In contrast, chronic HBV infection is characterized by weak or undetectable HBV-specific CD8^+^ T cell responses and the presence of functionally exhausted HBV-specific CD8^+^ T cells that are unable to clear the virus (45). We have previously reported that murine IFNα4 and IFNα5 treatment could strongly increase the activation, cytotoxic capacity, and cytokine production of both NK cells and CD8^+^ T cells in an HBV HDI mouse model which mimics acute-resolving HBV infection (46). However, anti-HBV innate and adaptive immune responses are not compromised in this mouse model (36), and it remained unclear whether these two IFNα subtypes could recover NK cell and T cell responses in the scenario of chronic HBV infection. Here, we demonstrate that murine IFNα4 and IFNα5 show only very limited effects in inducing NK cell and T cell activation in the rAAV8-HBV1.3 chronic infection mouse model. Instead, we identified murine IFNα2 as the most efficient subtype in inducing NK cell and CD8^+^ T cell activation among all tested IFNα subtypes in chronic HBV-infected mice. In addition to the different time points of HBV infection (acute versus chronic), there are several other substantial discrepancies between our previous study (46) and the current study. Firstly, the HBV HDI mouse model is not an infection model and the HBV replication is limited to the hepatocytes, that were initially transfected with the respective plasmid. In contrast, the newly generated rAAV8-HBV1.3 recombinant virus can constantly infect mouse hepatocytes, which better mimics the process of a natural HBV infection in humans. Secondly, the 10-days regime of IFNα subtype treatment in the previous study started 1 day prior to HBV HDI, suggesting that cells become alerted toward an antiviral state and antiviral effectors can already be expressed before viral entry. In contrast, in the current study we chose to treat mice with IFNα subtypes 4 weeks after rAAV8-HBV1.3 inoculation, when persistent HBV infection is already established. The latter model should better mimic the clinical scenario of treating CHB patients as HBV HDI. In clinical practice, human IFNα2 is the only available subtype for the treatment of chronic HBV (CHB) patients so far. Pegylated human IFNα2 has been shown to have both direct antiviral and immunomodulatory effects in CHB patients and it is conceivable that treatment outcome may be mostly triggered by the immunomodulatory effects of peg-IFNα2 on the innate and adaptive immune responses (47). For example, treatment with IFNα2 in CHB patients has been shown to significantly enhance NK cell frequency and their antiviral effector cytokine production (48, 49). Very recently, we have reported that patients who received IFNα treatment demonstrated a more active phenotype of global T cells than IFN–naïve patients, although no significant increase in HBcAg-specific CD8^+^ T cell responses were found in patients who received IFNα treatment compared to those without (50). In this study, we demonstrated that different human IFNα subtypes may also differ in their abilities to modulate T cell responses in healthy individuals. Future studies are needed to characterize the capacity of different human IFNα subtypes to improve anti-HBV NK and T cell responses in CHB patients.

In addition to activating the antiviral cellular immune response, IFNs could also mediate their antiviral effects through the transcriptional regulation of relevant genes, such as ISGs (51). IFNα induces several hundred ISGs, and a number of ISGs like MxA, APOBEC3G, MyD88, ISG20 and TRIM22 have been reported as effectors that actively inhibit transcriptional and post-transcriptional HBV gene expression (52–54). We recently reported that human IFNα14 is the most effective subtype in suppression of HBV cccDNA transcription and HBeAg/HBsAg production in HBV infected human cell lines *in vitro* as well as humanized mice *in vivo* (18). In particular, the induction of the restriction factor GBP5 by IFNα14 seemed to be required for HBV control (18). In previous studies in HIV-infected humanized mice and HIV-infected PBMCs and gut-derived mononuclear cells, as well as PBMCs from HIV-infected patients different immunomodulatory effects of human IFNα subtypes were observed with IFNα2 mainly modulating T cell responses, whereas IFNα14 stimulation prevented hyperimmune activation of T cells and improved cytotoxic NK cell responses (20, 55, 56). So far, it remains unclear whether human IFNα14 is also efficient in inducing anti-HBV NK and T cell responses, and more important, whether therapeutic combinations of different IFNα subtypes are effective in inducing antiviral ISGs and anti-HBV NK and T cell responses, which together might achieve a synergistic effect in suppressing HBV replication. Further studies are needed to address this issue and to develop more efficient treatment strategies for chronic hepatitis B therapy.

## Data availability statement

The original contributions presented in the study are included in the article/**Supplementary Material**. Further inquiries can be directed to the corresponding authors.

## Ethics statement

The studies involving human participants were reviewed and approved by Ethics Committee of the University Hospital Essen. The patients/participants provided their written informed consent to participate in this study. The animal study was reviewed and approved by Experimental Animal Ethics Committee, Tongji Medical College, Huazhong University of Science and Technology.

## Author contributions

KS and JL conceived the study. XX and ZK substantially contributed to the acquisition and analysis of the data. DA, JS, JI, XZ, XF, and XY contributed to sample preparation and performed experiments. DY and UD contributed to the interpretation of the results. KS and JL wrote the original manuscript. All authors contributed to the article and approved the submitted version.

## Funding

This work was supported by the National Natural Science Foundation of China (81861138044, 82172256, 92169105) to JL, and the DFG SU1030/1 to KS. This project was supported by the Sino-German Virtual Institute for Viral Immunology (SGVIVI). We acknowledge support by the Open Access Publication Fund of the University of Duisburg-Essen and the Laboratory Animal Center, Huazhong University of Science and Technology.

## Conflict of interest

The authors declare that the research was conducted in the absence of any commercial or financial relationships that could be construed as a potential conflict of interest.

## Publisher’s note

All claims expressed in this article are solely those of the authors and do not necessarily represent those of their affiliated organizations, or those of the publisher, the editors and the reviewers. Any product that may be evaluated in this article, or claim that may be made by its manufacturer, is not guaranteed or endorsed by the publisher.

## Supplementary material

Supplementary Figure 1 Virological and biochemical parameters after IFN treatment. Male C57BL/6 mice were intravenously injected with rAAV8-1.3HBV to establish HBV infection. After 29 days post infection mice were treated by intraperitoneal injection with 8000 U mIFNα subtypes for 10 consecutive days. Blood samples were collected before, during, and after mIFN treatment to dynamically monitor the characteristics of serum viremia. The day after the end of treatment with mIFNα subtype, the mice were sacrificed to freshly separate the intrahepatic lymphocytes for immune function analysis by flow cytometry. For a second group of mice the same procedure was additionally repeated at 50 dpi. **(A)** The HBsAg **(B)** and HBeAg levels after IFNα subtypes administration were shown (ctrl, n=4-7; IFNα2, n=2-6; IFNα4, n=5-10; IFNα5, n=5-10; IFNα11, n=5-9). **(C)** The ALT **(D)** and AST levels after IFNα subtypes administration were shown (ctrl, n=4; IFNα2, n=2-5; IFNα4, n=5; IFNα5, n=5; IFNα11, n=5). Statistical analyses between the treated groups and the untreated group were done by one-way ANOVA *p<0.05; **p<0.01.

Supplementary Figure 2 Gating strategy for liver and spleen infiltrating lymphocytes. Flow cytometry gating scheme for monocytes, dendritic cells, NK cells and CD8^+^ T cells are shown.

Supplementary Figure 3 Immunomodulatory activities of different IFNα subtypes during HBV infection in spleen and liver. Male C57BL/6 mice were intravenously injected with rAAV8-1.3HBV to establish HBV infection. After 29 days post infection mice were treated by intraperitoneal injection with 8000 U mIFNα subtypes for 10 consecutive days. Blood samples were collected before, during, and after mIFN treatment to dynamically monitor the characteristics of serum viremia. The day after the end of treatment with mIFNα subtype, the mice were sacrificed to freshly separate the intrahepatic lymphocytes and splenocytes for immune function analysis by flow cytometry. For a second group of mice the same procedure was additionally repeated at 50 dpi. The cell number of infiltrating lymphocytes in liver **(A)** and spleen **(D)** were shown. The frequency of NK cells and T cells of infiltrating lymphocytes in liver **(B, C)** and spleen **(E)** and **(F)** were shown. **(G–P)** The phenotypes and effector function analysis of monocyte/dendritic cell/NK and HBV-specific CD8 T cell in spleen were shown. Individual mice are depicted as dots. Mean values ± SEM are shown for 1x ctrl, n=3; 1x IFNα2, n=3; 1x IFNα4, n=5; 1x IFNα5, n=5; 1x IFNα11, n=4; 2x ctrl, n=3-5; 2x IFNα2, n=4-5; 2x IFNα4, n=5; 2x IFNα5, n=5; 2x IFNα11, n=5. Statistical analyses between the treated groups and the untreated group were done by one-way ANOVA *p<0.05; **p<0.01; ***p<0.001; ****p<0.0001.

Supplementary Figure 4 Comparison of intrahepatic immune responses in naïve and chronically infected mice after IFNα2 treatment. **(A)** Naïve male C57BL/6 mice were treated by intraperitoneal injection with 8000 U mIFNα subtypes for 10 consecutive days. One day after the treatment with mIFNα subtype, the mice were sacrificed to freshly separate the intrahepatic lymphocytes for immune function analysis by flow cytometry. Created with BioRender.com. **(B, C)** The MHCI expression (MFI) of intrahepatic monocytes and dendritic cells was analyzed by flow cytometry. **(D–F)** Frequencies of CD69/GzmB/IFNγ expressing intrahepatic NK cells and **(G–I)** CD8^+^ T cells were analyzed by flow cytometry. Individual mice are depicted as dots. Mean values ± SEM are shown for ctrl, 3-5; IFNα2, n=5-6; IFNα4, n=6; IFNα5, n=3-6; IFNα11, n=4-6 Statistical analyses between the treated groups and the untreated group were done by one-way ANOVA. *p<0.05; **p<0.01; ***p<0.001.
