# Supplementary figures and images for: IFNα subtype-specific susceptibility of HBV in the course of chronic infection
Source: Front Immunol. 2022 Oct 14;13:1017753. doi: 10.3389/fimmu.2022.1017753 (PMC9616162; doi:10.3389/fimmu.2022.1017753)

**A**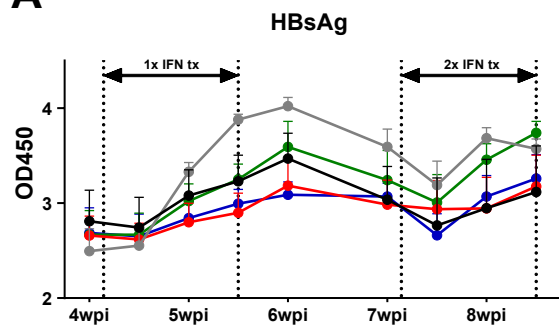**B**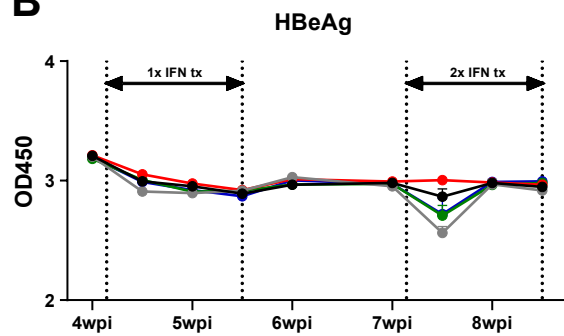**C**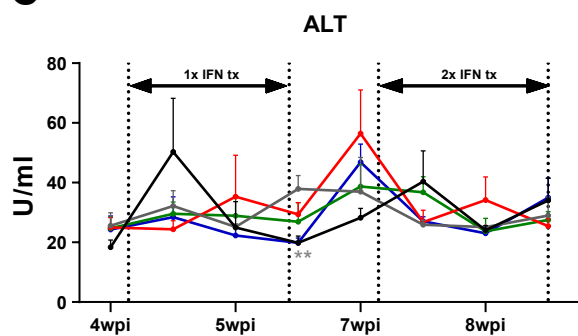**D**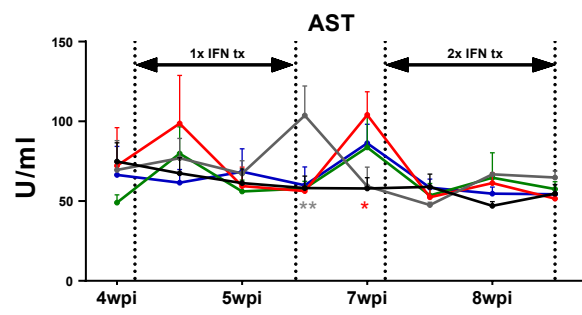

○ ctrl      ● IFNα2      ● IFNα4      ● IFNα5      ● IFNα11

Supplement: Supplementary Figure 1 — Virological and biochemical parameters after IFN treatment. Male C57BL/6 mice were intravenously injected with rAAV8-1.3HBV to establish HBV infection. After 29 days post infection mice were treated by intraperitoneal injection with 8000 U mIFNα subtypes for 10 consecutive days. Blood samples were collected before, during, and after mIFN treatment to dynamically monitor the characteristics of serum viremia. The day after the end of treatment with mIFNα subtype, the mice were sacrificed to freshly separate the intrahepatic lymphocytes for immune function analysis by flow cytometry. For a second group of mice the same procedure was additionally repeated at 50 dpi. (A) The HBsAg (B) and HBeAg levels after IFNα subtypes administration were shown (ctrl, n=4-7; IFNα2, n=2-6; IFNα4, n=5-10; IFNα5, n=5-10; IFNα11, n=5-9). (C) The ALT (D) and AST levels after IFNα subtypes administration were shown (ctrl, n=4; IFNα2, n=2-5; IFNα4, n=5; IFNα5, n=5; IFNα11, n=5). Statistical analyses between the treated groups and the untreated group were done by one-way ANOVA *p<0.05; **p<0.01. [file Image_1.pdf]

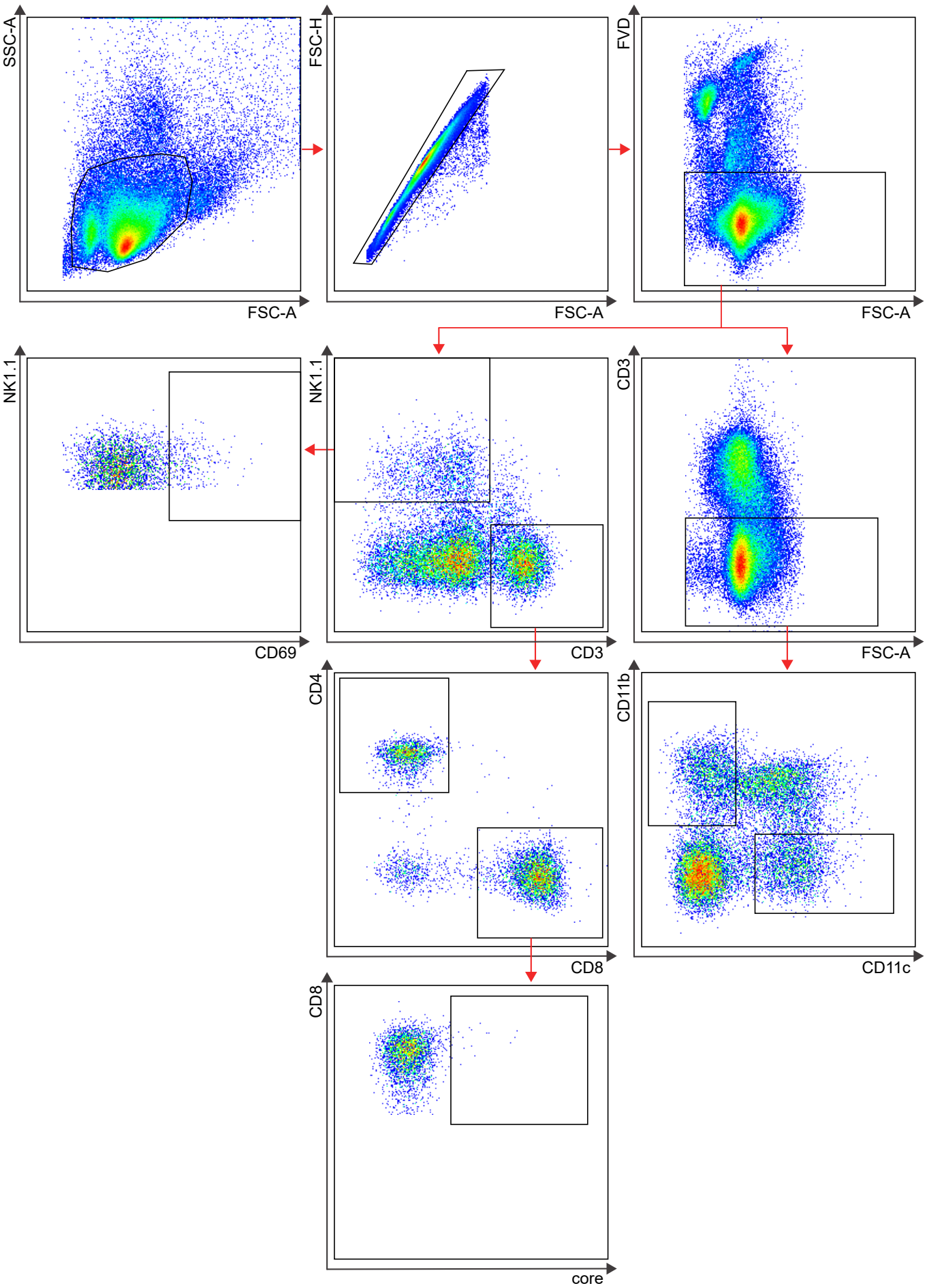

Supplement: Supplementary Figure 2 — Gating strategy for liver and spleen infiltrating lymphocytes. Flow cytometry gating scheme for monocytes, dendritic cells, NK cells and CD8+ T cells are shown. [file Image_2.pdf]

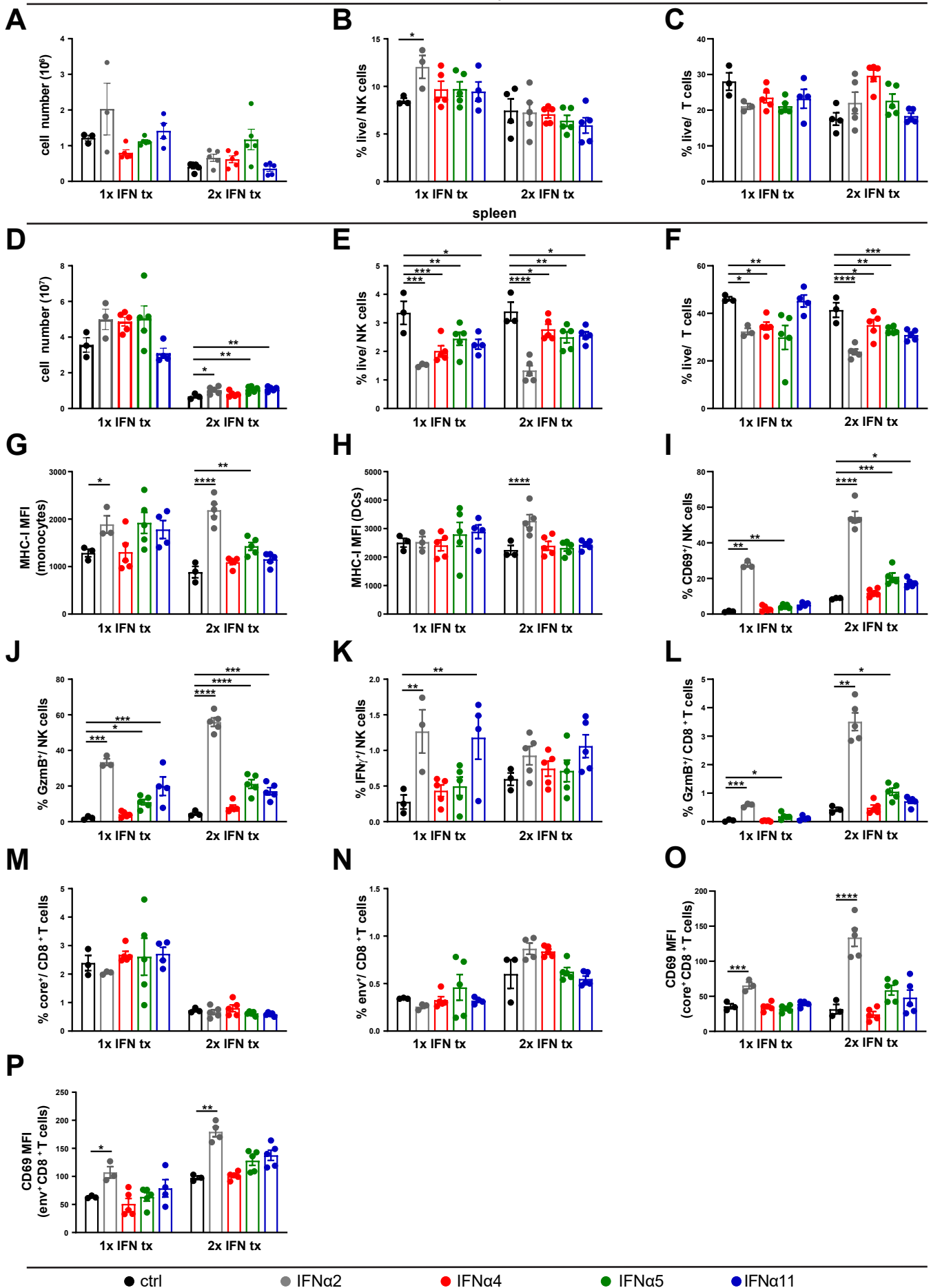

Supplement: Supplementary Figure 3 — Immunomodulatory activities of different IFNα subtypes during HBV infection in spleen and liver. Male C57BL/6 mice were intravenously injected with rAAV8-1.3HBV to establish HBV infection. After 29 days post infection mice were treated by intraperitoneal injection with 8000 U mIFNα subtypes for 10 consecutive days. Blood samples were collected before, during, and after mIFN treatment to dynamically monitor the characteristics of serum viremia. The day after the end of treatment with mIFNα subtype, the mice were sacrificed to freshly separate the intrahepatic lymphocytes and splenocytes for immune function analysis by flow cytometry. For a second group of mice the same procedure was additionally repeated at 50 dpi. The cell number of infiltrating lymphocytes in liver (A) and spleen (D) were shown. The frequency of NK cells and T cells of infiltrating lymphocytes in liver (B, C) and spleen (E) and (F) were shown. (G–P) The phenotypes and effector function analysis of monocyte/dendritic cell/NK and HBV-specific CD8 T cell in spleen were shown. Individual mice are depicted as dots. Mean values ± SEM are shown for 1x ctrl, n=3; 1x IFNα2, n=3; 1x IFNα4, n=5; 1x IFNα5, n=5; 1x IFNα11, n=4; 2x ctrl, n=3-5; 2x IFNα2, n=4-5; 2x IFNα4, n=5; 2x IFNα5, n=5; 2x IFNα11, n=5. Statistical analyses between the treated groups and the untreated group were done by one-way ANOVA *p<0.05; **p<0.01; ***p<0.001; ****p<0.0001. [file Image_3.pdf]

**A**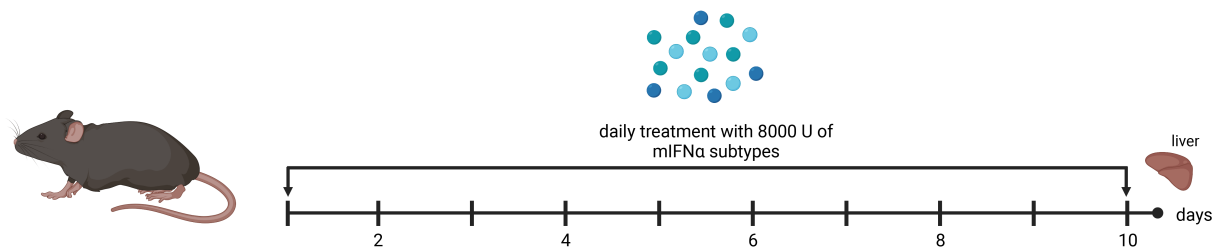**B**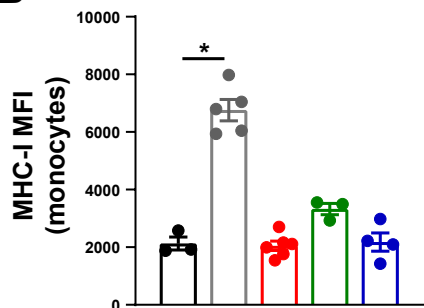**C**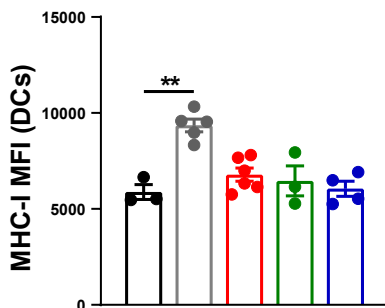**D**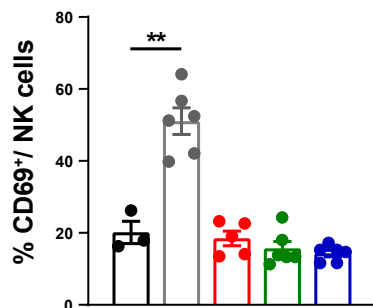**E**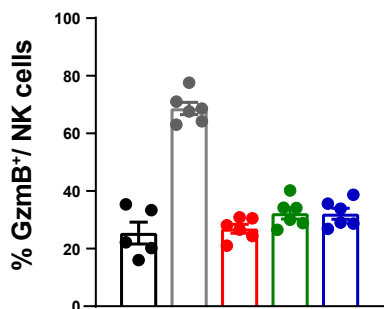**F**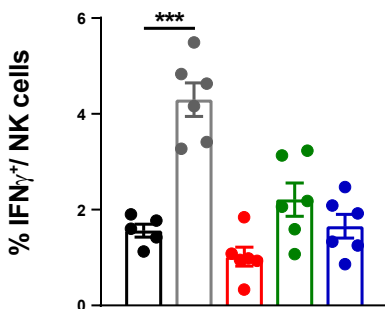**G**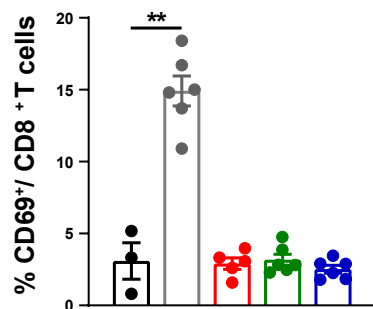**H**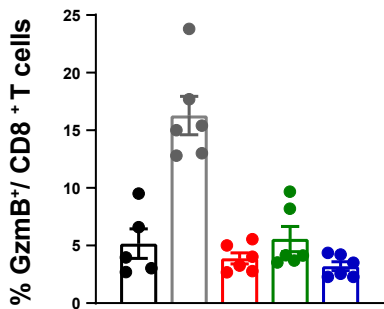**I**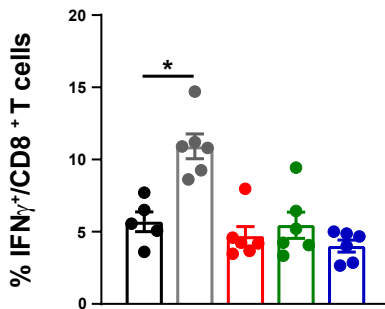

● ctrl

● IFNα2

● IFNα4

● IFNα5

● IFNα11

Supplement: Supplementary Figure 4 — Comparison of intrahepatic immune responses in naïve and chronically infected mice after IFNα2 treatment. (A) Naïve male C57BL/6 mice were treated by intraperitoneal injection with 8000 U mIFNα subtypes for 10 consecutive days. One day after the treatment with mIFNα subtype, the mice were sacrificed to freshly separate the intrahepatic lymphocytes for immune function analysis by flow cytometry. Created with BioRender.com. (B, C) The MHCI expression (MFI) of intrahepatic monocytes and dendritic cells was analyzed by flow cytometry. (D–F) Frequencies of CD69/GzmB/IFNγ expressing intrahepatic NK cells and (G–I) CD8+ T cells were analyzed by flow cytometry. Individual mice are depicted as dots. Mean values ± SEM are shown for ctrl, 3-5; IFNα2, n=5-6; IFNα4, n=6; IFNα5, n=3-6; IFNα11, n=4-6 Statistical analyses between the treated groups and the untreated group were done by one-way ANOVA. *p<0.05; **p<0.01; ***p<0.001. [file Image_4.pdf]
