# Supplementary material for: IFNα subtype-specific susceptibility of HBV in the course of chronic infection
Source: Front Immunol. 2022 Oct 14;13:1017753. doi: 10.3389/fimmu.2022.1017753 (PMC9616162; doi:10.3389/fimmu.2022.1017753)
Supplement: Supplementary file 5 [file Table_1.pdf]

Supporting Information

Supplementary Table 1: Used antibodies

Target	clone	company
anti-hu/ms-GzmB	GB11	BD Bioscience
anti-hu-CD107a	H4A3	BioLegend
anti-hu-CD28	9F10	BioLegend
anti-hu-CD3	UCHT1	eBioscience™
anti-hu-CD38	HIT2	BD Bioscience
anti-hu-CD4	RPA-T4	BioLegend
anti-hu-CD49d	CD2.2	BioLegend
anti-hu-CD8	RPA-T8	BioLegend
anti-hu-IFNγ	B27	BioLegend
anti-ms-CD11b	M1/70	BioLegend
anti-ms-CD11c	N418	BD Bioscience
anti-ms-CD3	145-2C11	BioLegend
anti-ms-CD4	GK1.5	BioLegend
anti-ms-CD8	53-6.7	BD Bioscience
anti-ms-IFNγ	XMG1.2	BioLegend
anti-ms-MHC-I	KH95	eBioscience
anti-ms-NK1.1	PK136	BD Bioscience
MHC Tetramer<br>H-2kb HBV core		MBL
MHC Tetramer<br>H-2kb HBV HBsAg		MBL

Supplementary Table 2: Primer sequences

Gene	Forward sequence (5´->3´)	Forward sequence (5´->3´)
b-actin	ggactcctatgtgggtgacgag	tcacggttggccttagggtt
Ifna1	atttcccctgacccaggaagatg	tcccagcacattggcagagg
Ifna2	ctgtgctttcctcgtgatgc	tcaaggccctcttgttcctg
Ifna4	aagagggccttgacagtcct	gggagtcttcctgggtcaga

Ifna5	gattcccacaggagaaggtgg	ccttgctcaatcttgccagc
Ifna6	ccctgaagatccagaaagaga	gtatctaggagggttgcatcc
Ifna7	acctcaggaacaagagagcct	catgcagaacacagagggcttg
Ifna8	atggctaggctctgtgcttt	gctcagtcaggacagggatg
Ifna9	ctggtcgggatgaaggaactg	tctcctcactcagtcttgcc
Ifna11	tgtgctgcgagatcttaccc	ctgggtcaggggagattcct
Ifna12	acctgcaaggctgtctgatg	agaatttgagcagtgagggtca
Ifna13	aggctcaagccatccctttt	agaagagtcctctccacact
Ifna14	aggaccggaaggactttggat	tcaggggaggtgcctgtatc
IfnaA	gctttcctgatgaccctgct	gctgggtcagctcttgtagg
IfnaB	attggcaatattcaccatggctagg	tctcctgggggaatccgaag
